# Diagnostic Potential of Volatile Organic Compounds in Detecting Insulin Resistance Among Taiwanese Women

**DOI:** 10.3390/diagnostics15141817

**Published:** 2025-07-18

**Authors:** Fan-Min Lin, Jin-Hao Xu, Chih-Hao Shen, Sheng-Tang Wu, Ta-Wei Chu

**Affiliations:** 1 Division of Pulmonary Medicine, Department of Internal Medicine, Kaohsiung Armed Forces General Hospital, Kaohsiung 802, Taiwan; glutamate31@hotmail.com (F.-M.L.); imchopper619@gmail.com (J.-H.X.); 2 Division of Pulmonary and Critical Care Medicine, Department of Medicine, Tri-Service General Hospital, National Defense Medical University, Taipei 114, Taiwan; potato652@yahoo.com.tw; 3 Division of Urology, Department of Surgery, Tri-Service General Hospital, National Defense Medical University, Taipei 114, Taiwan; doc20283@gmail.com; 4 Division of Urology, Department of Surgery, Kaohsiung Armed Forces General Hospital, Kaohsiung 802, Taiwan; 5 Department of Obstetrics and Gynecology, Tri-Service General Hospital, National Defense Medical University, Taipei 114, Taiwan; 6 MJ Health Research Foundation, Taipei 114, Taiwan

**Keywords:** volatile organic compound, insulin resistance, machine learning, Taiwanese women

## Abstract

**Background**: Insulin resistance (IR) is an underlying pathophysiology for type 2 diabetes (T2D). The Homeostasis Model Assessment of Insulin Resistance (HOMA-IR) is the simplest method for evaluating IR. At the same time, volatile organic compounds (VOCs) detected in human respiration can be correlated with specific diseases. To date, machine learning (Mach-L) has yet to be used to examine potential relationships between VOCs and IR. The present study has two aims: (1) to identify the VOCs most relevant to HOMA-IR, and (2) to use Shapley addictive explanation (SHAP) to determine the impacts of the distributions and directions of each feature in Taiwanese women. **Methods:** A total of 1432 Taiwanese women between the ages of 19 and 84 years were enrolled, and 344 VOCs were measured. Traditional multiple linear regression (MLR) was used as a benchmark for comparison, applying three Mach-L methods. Finally, SHAP was used to evaluate the directions of impacts of the features on HOMA-IR. **Results**: Six VOCs were identified as important: dimethylfuran, propanamine, aniline, butoxyethanol, and isopropyltoluene, in order from most to least important. SHAP found that dimethylfuran, isopropyltoluene, and dodecane were positively correlated to HOMA-IR, while butoxyethanol, aniline, and propanamine were negatively correlated. **Conclusions**: Using three different Mach-L methods, six VOCs were selected to be related to IR in Taiwanese women. According to their importance, dimethylfuran, propanamine, aniline, butoxyethanol, and isopropyltoluene could be used to help diagnose HOMA-IR. Furthermore, by using SHAP, dimethylfuran, isopropyltoluene, and dodecane had a positive and the other three had a negative influence.

## 1. Introduction

Type 2 diabetes (T2D) is a major global health concern. In 2023, approximately 529 million individuals were living with T2D, which is projected to increase to 1.3 billion by 2050 [1,2]. Taiwan has seen a similar trend, with around 11.8% of its population affected by T2D [3], up from 10.0% in 2015 and 10.9% in 2017. T2D is currently the fifth leading cause of death in Taiwan and, along with related complications, accounts for 11.5% of outlays by that country’s national health insurance program. Thus, the early detection and management of T2D are urgent challenges both in Taiwan and worldwide.

The pathophysiology of T2D is complex, but it is widely recognized that insulin resistance (IR) and β-cell dysfunction are key contributors [4,5]. IR can be assessed through various methods, with the hyperinsulinemic euglycemic clamp considered the gold standard [6]. However, these methods are costly and labor-intensive. In contrast, the Homeostasis Model Assessment of Insulin Resistance (HOMA-IR), developed by Matthews et al., offers a simpler way to assess IR by using measurements of fasting plasma glucose (FPG) and fasting plasma insulin (FPI) [7]. Due to its ease of use, HOMA-IR is commonly used in research studies.

At the same time, for more than fifty years, researchers have explored the potential diagnostic value of volatile organic compounds (VOCs) emitted by the human body. In 1971, Linus Pauling demonstrated that human respiration contains a complex mixture of around 250 VOCs [8]. These compounds are generated through metabolic processes, transported to the lungs via the bloodstream, and subsequently exhaled. Changes in the concentrations of these exhaled compounds can be directly correlated with specific diseases, including cancer [9]. Techniques such as gas chromatography–mass spectrometry (GC-MS) have been used to effectively establish connections between VOCs and various diseases [10,11,12,13,14]. Although numerous studies have analyzed VOCs data to identify diseases, few have investigated the application of machine learning (Mach-L) techniques for VOCs profiling [15,16]. The present study seeks to use VOCs to help detect HOMA-IR, and has three distinct aims:

1. To compare the performance of traditional multiple linear regression (MLR) and Mach-L.
2. To use the Mach-L methods to identify the VOCs that are most relevant to HOMA-IR.
3. To use Shapley addictive explanation (SHAP) to determine the impacts of distributions and directions of each feature in Taiwanese women.

## 2. Materials and Methods

### 2.1. Participants and Study Design

**Figure 1:**
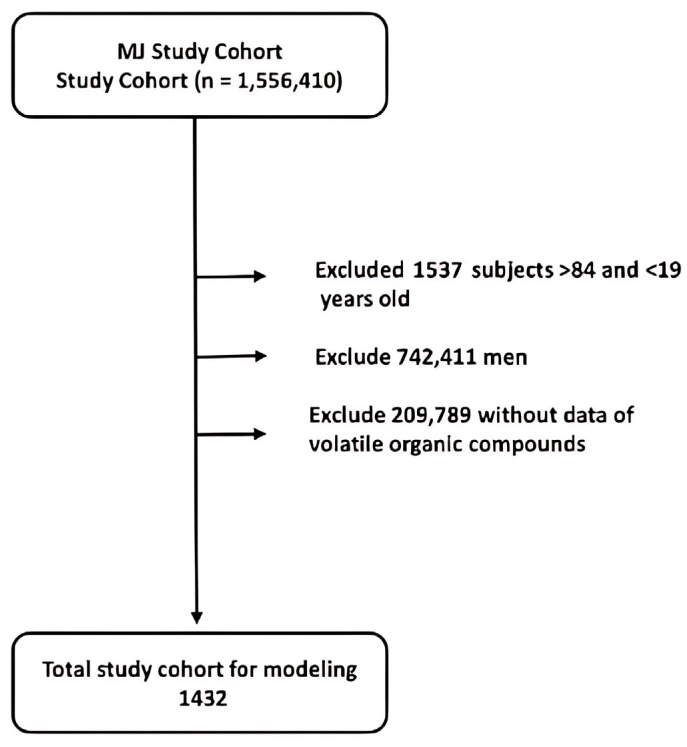
Participant selection scheme.

The present study followed our previously published protocol [17]. Data were sourced from the Taiwan MJ cohort, an ongoing prospective cohort of health examinations conducted by the MJ Health Screening Centers in Taiwan [18]. These examinations cover more than 100 important biological indicators, including anthropometric measurements, blood tests, imaging tests, etc. Each participant completed a self-administered questionnaire to collect information related to personal and family medical history, current health status, lifestyle, physical exercise, sleep habits, and dietary habits [19]. It should be noted that this study is a secondary data analysis. MJ Clinic patients provided general consent for future anonymous studies. All or part of the data used in this research was authorized by and received from MJ Health Research Foundation (Authorization Code: MJHRF2022010A). Any interpretations or conclusions described in this paper do not represent the views of MJ Health Research Foundation. For additional information, please refer to the Foundation’s annual technical report [19]. This study’s protocol was approved by the Institutional Review Board of the Kaohsiung Armed Forces General Hospital (IRB No.: KAFGHIRB 111-011). Since no samples were collected from patients, a short IRB review was approved, and no consent was required. The dataset initially included 1,556,410 healthy participants. Filtering for the inclusion criteria listed below, the final sample for analysis included 1432 women (see Figure 1). A total of 344 VOCs were measured (see Appendix A, Table A1).

Sample inclusion criteria are as follows:

1. Age between 19 and 84 years old (included).
2. Women without significant medical diseases.
3. Availability of all VOCs values and other demographic, biochemistry, and lifestyle information.

For the sake of brevity, please refer to our previous study [17] for details of the methods used for measuring demographics and biochemistry. We further divided our participants into two groups, i.e., with and without IR. The cut-off point for having IR was >1.78 μU × mg/mL/mL × dL [20].

Table 1 summarizes the 19 demographic, biochemistry, and lifestyle (dependent) variables, while HOMA-IR is the independent variable, calculated as follows:

$$HOMA-IR=\frac{FPI(\mu U/mL)\times FPG(mg/dL)}{405}$$

### 2.2. Traditional Statistics

To compare HOMA-IR performance using different demographic, biochemistry, and lifestyle parameters, we used *t*-tests to compare HOMA-IR for marital status, and analyzed variances to compare the HOMA-IR in terms of ordinal data such as education and income level. We used the Kolmogorove–Smirnov test to evaluate the normality assumption and variance inflation factor to validate the multicollinearity assumption. A simple correlation was applied to evaluate the relationship between HOMA-IR and other parameters. MLR was also used to set a benchmark for comparison among the various Mach-L methods. In order to further investigate the relationships between all parameters and HOMA-IR, we applied logistic regression. In this analysis, participants were divided into two groups: IR and without IR. We coded participants without IR as 0, and those with IR as 1, converting the original continuous variable into a categorical one. Logistic regression provided information such as the area under the receiver operating characteristic (ROC) curve, sensitivity, specificity, positive predictive value (PPV), and negative predictive value (NPV). We performed logistic regression twice: first using only VOCs as independent variables, and then including additional features such as demographic, biochemical, and lifestyle factors. The aforementioned tests were performed using SPSS version 19.0 (IBM Inc., Armonk, NY, USA).

### 2.3. Protocol for Breath Sample Collection

All participants were asked to remain motionless for 10 min and to rinse their mouths with unchlorinated water before breathing through a mouthpiece fitted with a three-way direct-connect valve. In the first stage, participants exhaled through the first egress connected to a gas bag (SKC Inc., Eighty-Four, PA, USA) for volume estimation. When the exhalation volume reached approximately 0.3 L, the valve was switched to the second egress, connected to a 1.0 L aluminum bag for late expiratory fraction collection for VOCs analysis. The collection process was repeated up to 3 times as necessary to collect sufficient samples for analysis. The sealed samples were kept at room temperature (25 °C) and analyzed within 48 h of collection. To ensure valid storage conditions, we performed a time-dependent analysis (twice each day for three days) on ten breath samples, the results of which showed that most VOCs remained stable over time.

### 2.4. VOCs Analysis Using SIFT-MS

A selected-ion flow-tube mass spectrometry (SIFT-MS, VOICE 200 ultra, Syft Technologies, Christchurch, New Zealand) was applied to VOCs in the collected late expiratory fraction. SIFT-MS analyzes VOCs in air or vapor samples by chemical ionization. The quadrupole mass spectrometer detects characteristic products derived from the ionization of VOCs in breath samples by injecting selected precursor ions (H_3_O^+^, NO^+^, and O_2_^+^) into the nitrogen carrier gas. The downstream detection system measures the count rate of both precursor ions and the characteristic product ions in real-time. Volatile compounds are measured in parts-per-billion or parts-per-million by volume. Multiple VOCs can be simultaneously quantified in a gaseous mixture. We selected more than 300 compounds for analysis, including alkanes, ketones, aldehydes, alcohols, amines, thiols, and others. Among these VOCs, some product ions overlap, for example, MH+ ions at 43, 47, 57, 59, 60, 61, 69, 71, 75, 85, 87, 89, 91, 97, 99, 101, and 103. The quantitative estimations of the selected VOCs were performed based on the pre-set SIFT-MS protocol. In this setting, several product ions are derived from three different reagent ions, H_3_O^+^, O_2_^+^, and NO^+^, with a tolerance setting of 20%. The tolerance feature is designed to deal with product ion interferences for all VOCs. The final analyte concentration is calculated as an average of the lowest product ion concentration and anything within 20%. Any ions beyond the 20% tolerance range are excluded from the calculation. Some VOCs suffering from interference from product ions cannot be resolved by adjusting the tolerance settings and can only be quantified on a relative basis. To this end, statistical models are constructed based on accurate measurements and the relative scales of the VOCs.

### 2.5. Mach-L Methods

This study constructs predictive models for MLR using three different Mach-L methods: stochastic gradient boosting (SGB), eXtreme Gradient Boosting (XGBoost), and Elastic net (EN). A detailed description of these methods can be found in our previous work [21].

**Figure 2:**
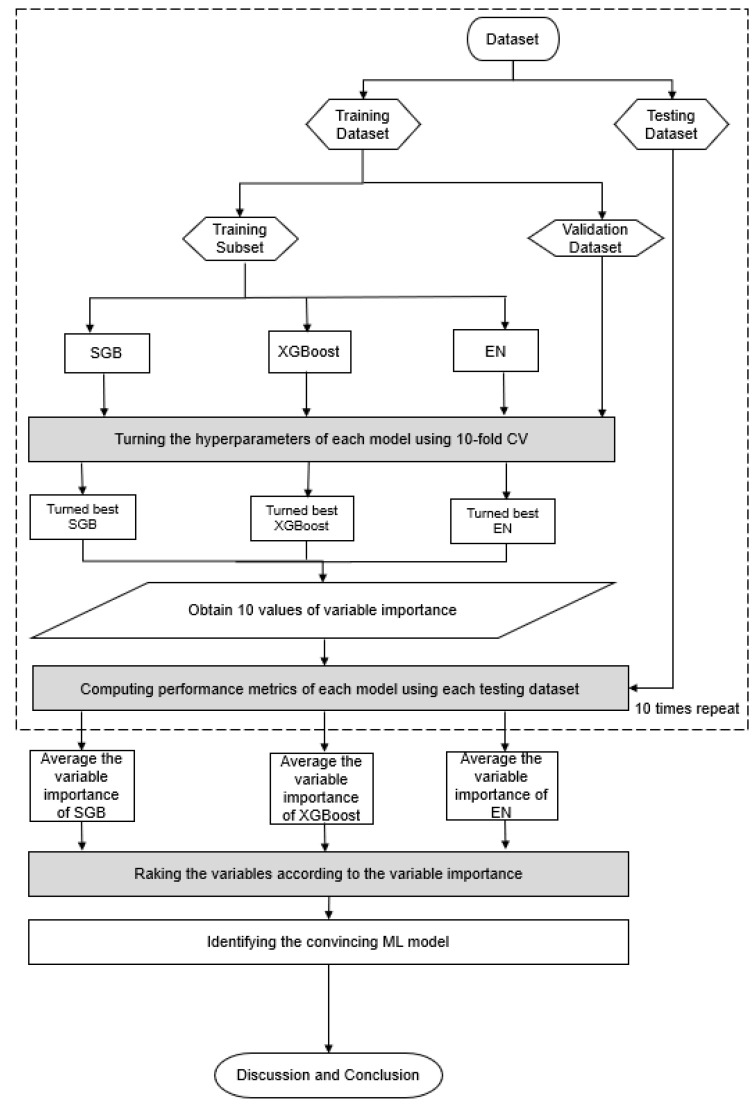
Flowchart of proposed scheme.

Figure 2 presents a flowchart of the proposed prediction and key variable identification scheme using the three Mach-L methods. Initially, patient data were collected and organized into the dataset using the proposed method. The dataset was then randomly divided into an 80% training dataset for model building and a 20% testing dataset for evaluation. During the training process, each Mach-L method was fine-tuned with specific hyperparameters to construct well-performing models using a 10-fold cross-validation technique. The training dataset was further partitioned into a training subset with a different set of hyperparameters and a validation subset for model validation. A grid search explored all possible hyperparameter combinations. The model with the lowest root mean square error for the validation subset was deemed the best model for each Mach-L method. The best-tuned SGB, XGBoost, and EN models were generated, and the corresponding variable importance ranking information was obtained.

In the evaluation phase, the testing dataset was used to gauge the predictive efficacy of the MARS model. Given that the target variable in this study is a numerical parameter, the evaluation metrics chosen as the basis for model performance comparison include mean absolute percentage error (MAPE), symmetric mean absolute percentage error (SMAPE), relative absolute error (RAE), root relative squared error (RRSE), and root mean squared error (RMSE) (see Table 2).

In this study, all methods were performed using R software version 4.0.5 and RStudio version 1.1.453 with the required packages installed [22,23]. The implementations of SGB, XGBoost, and EN were, respectively, the “randomForest” R package version 4.6-14 [24], “gbm” R package version 2.1.8 [25], “rpart” R package version 4.1-15 [26], and “XGBoost” R package version 1.5.0.2 [27]. In addition, to estimate the best hyperparameter set for the developed effective SGB, XGBoost, and EN methods, the “caret” R package version 6.0–90 was used [28]. The MLR was implemented using the “stats” R package version 4.0.5, and the default setting was used to construct the models.

SHAP was conducted using the following Python packages 0.41.0: SHAP, the core package for computing and visualizing SHAP values, provides interpretability for model predictions and feature importance. Pandas, a powerful library for data manipulation and preprocessing, was used to manage datasets, clean data, and prepare inputs for SHAP analysis. NumPy, a fundamental package for numerical computations, supports array operations and numerical calculations required by SHAP. Matplotlib v3.10.2, a plotting library for creating static, interactive, and animated visualizations, was used to generate SHAP plots, including summary plots, and bar plots. This feature contributions to specific predictions.

Finally, to justify SHAP’s interpretability accuracy, we chose the SHAP KernelExplainer which is one of the model-agnostic SHAP.

## 3. Results

Table 1 summarizes the demographic, biochemistry, and lifestyle information of the study cohort. As mentioned in the Methods Section, we further divided the study cohort into two groups: with and without IR. The results of the comparison of demographic, biochemical, and lifestyle information are shown in Table 3. It is worth noting that, except for estimated glomerular filtration rate, alcohol consumption, tobacco consumption, marital status, and income status, all other features were significantly different. However, this was not the main focus of our study.

**Figure 3:**
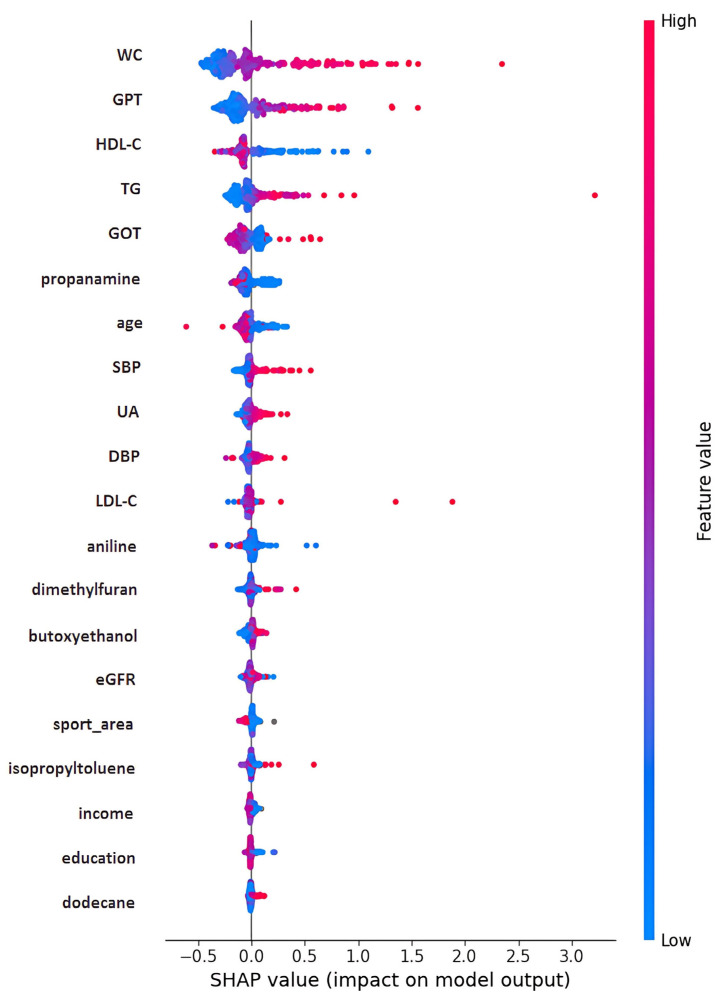
Bee swarm of SHAP. Note: SHAP, Shapley addictive explanation; WC, waist circumference; GPT, serum glutamic pyruvic transaminase; HDL-C, high-density lipoprotein cholesterol; TG, triglycerides; GOT, serum glutamic oxaloacetic transaminase; SBP, systolic blood pressure; UA, uric acid; DBP, diastolic blood pressure; LDL-C, low-density lipoprotein cholesterol; eGFR, estimated Glomerular filtration rate.

**Figure 4:**
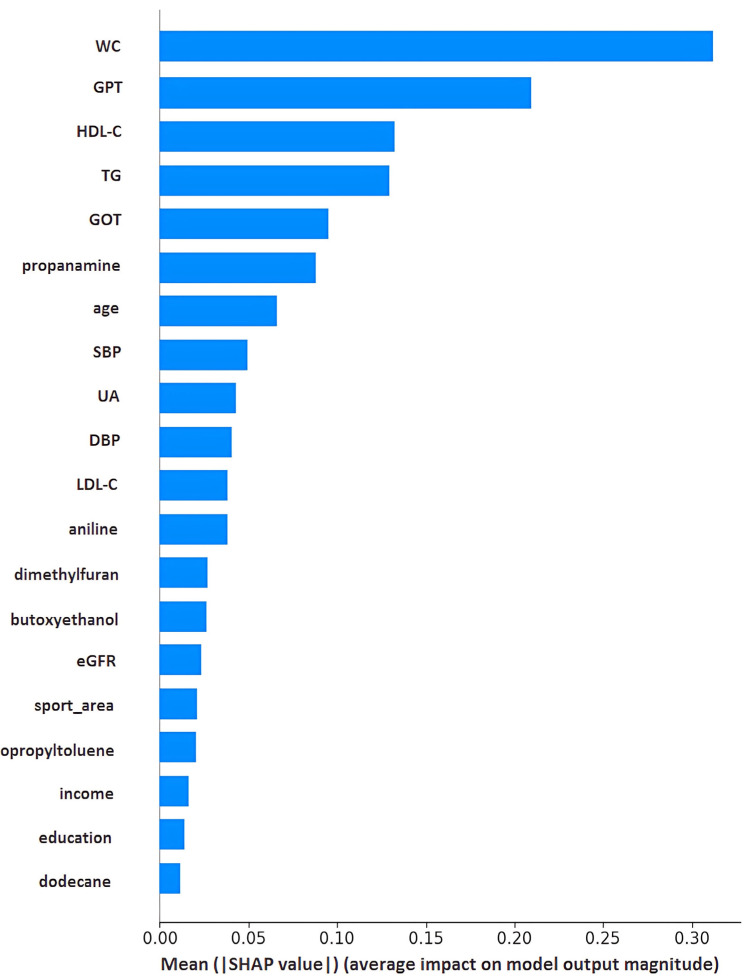
Absolute SHAP values of the features for stochastic gradient boosting. Note: SHAP, Shapley addictive explanation; WC, waist circumference; GPT, serum glutamic pyruvic transaminase; HDL-C, high-density lipoprotein cholesterol; TG, triglycerides; GOT, serum glutamic oxaloacetic transaminase; SBP, systolic blood pressure; UA, uric acid; DBP, diastolic blood pressure; LDL-C, low-density lipoprotein cholesterol; eGFR, estimated Glomerular filtration rate.

**Figure 5:**
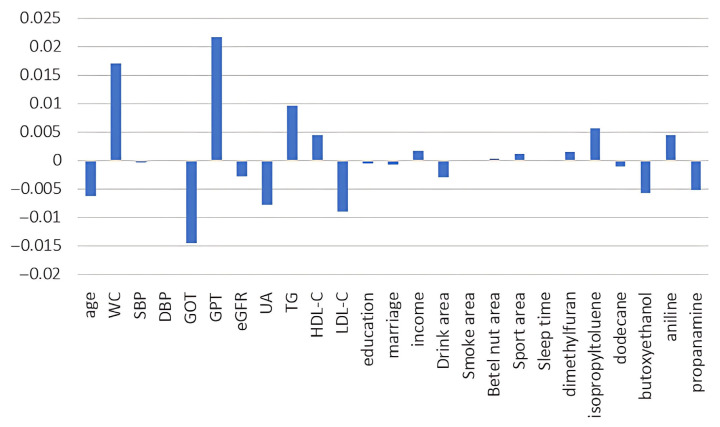
Net values of SHAP of the features for stochastic gradient boosting. Note: SHAP, Shapley addictive explanation; WC, waist circumference; SBP, systolic blood pressure; DBP, diastolic blood pressure; GOT, serum glutamic oxaloacetic transaminase; GPT, serum glutamic pyruvic transaminase; eGFR, estimated Glomerular filtration rate; UA, uric acid; TG, triglycerides; HDL-C, high-density lipoprotein cholesterol; LDL-C, low-density lipoprotein cholesterol.

**Figure 6:**
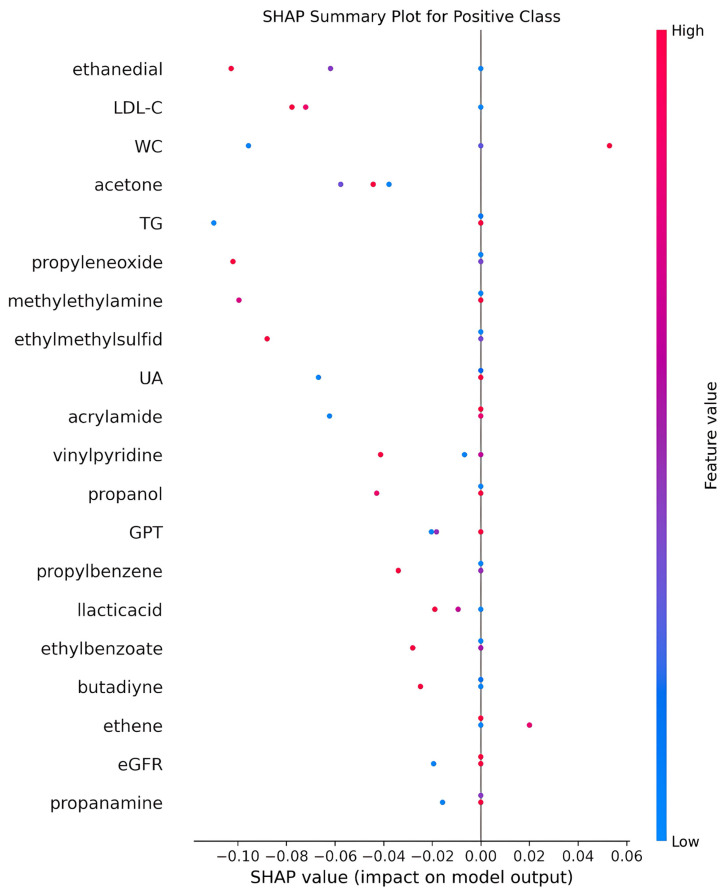
Summary plot of SHAP KernelExplainer for stochastic gradient boosting. Note: SHAP, Shapley addictive explanation; LDL-C, low-density lipoprotein cholesterol; WC, waist circumference; TG, triglycerides; UA, uric acid; GPT, serum glutamic pyruvic transaminase; eGFR, estimated Glomerular filtration rate.

The simple correlation results are presented in Table 4. HOMA-IR is found to be positively correlated with age, waist circumference (WC), glutamic oxaloacetic transaminase (GOT), glutamic pyruvic transaminase (GPT), uric acid (UA), triglyceride (TG), low-density lipoprotein cholesterol (LDL-C), and blood pressure, while negatively correlated with high-density lipoprotein cholesterol (HDL-C). None of the VOCs reached statistical significance. However, dimethylfuran, isopropyltoluene, and dodecane were positively related to HOMA-IR, while butoxyethanol, aniline, and propanamine were negatively related to HOMA-IR. To demonstrate the outperformance of the Mach-L methods, the errors of estimation are shown in Table 5. Our results demonstrated that all the estimation errors were smaller for the Mach-L methods than those of the MLR, indicating that the estimations of the Mach-L methods were more accurate. Table 6 shows the percentage of importance for all the features, including the six most important VOCs. The most important variable was WC, followed by TG, HDL-C, and liver enzymes. At the same time, the orders for the VOCs were dodecane, dimethylfuran, propanamine, aniline, butoxyethanol, and isopropyltoluene. The SHAP results are shown in Figure 3. Each of the three Mach-L methods has its own SHAP. We chose the SHAP of XGBoost to demonstrate the importance and direction of each feature and participant. This provides additional insight into the relationships between various features and HOMA-IR. To determine the overall impact of a feature, Figure 4 averages the absolute SHAP values from Figure 3, helping clinicians prioritize the features to be focused on. Since the absolute values of the SHAP do not give us a ‘direction’, Figure 5 shows the net values of SHAP (not the absolute values) reflecting the direction of the impacts for these features. Dimethylfuran, isopropyltoluene, aniline, and dodecane were positively correlated to HOMA-IR, while butoxyethanol and propanamine were negatively correlated to HOMA-IR. To justify SHAP’s interpretability accuracy, Figure 6 shows the summary plot of SHAP KernelExplainer.

**Figure 7:**
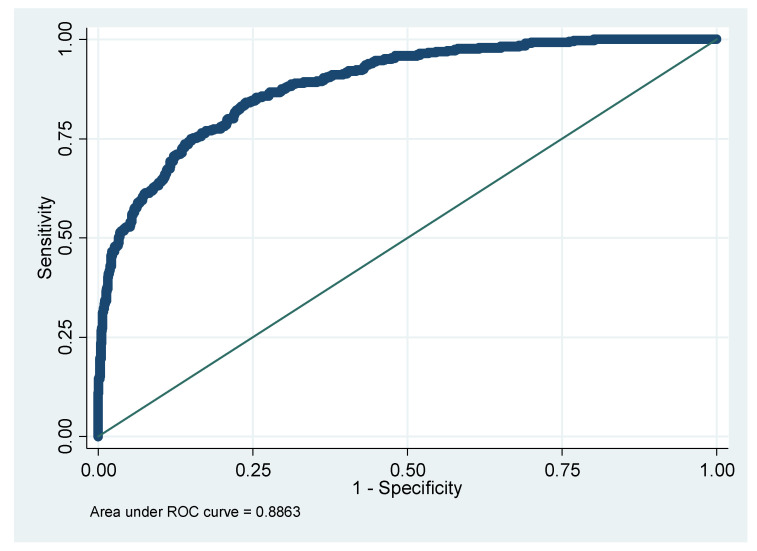
AUC for whether to have IR with only VOCs. Note: AUC, the area under the ROC curve; IR, insulin resistance; VOCs, volatile organic compounds.

**Figure 8:**
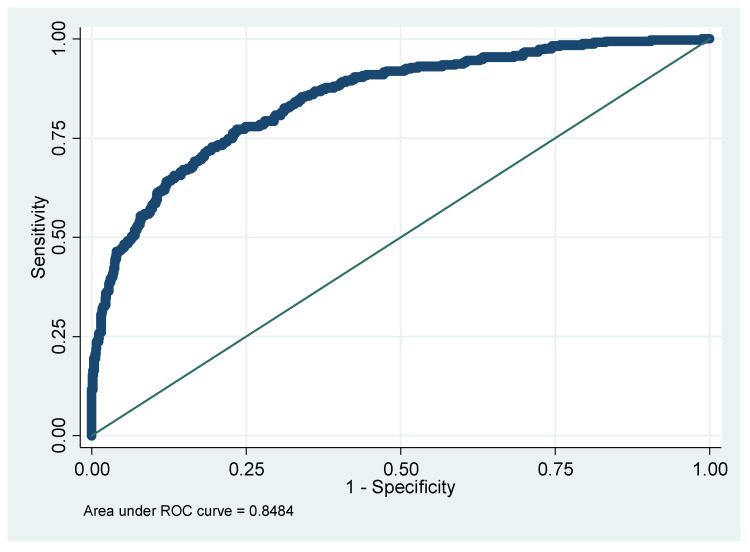
AUC for whether to have IR with demographic biochemistry and lifestyle model. Note: AUC, the area under the ROC curve; IR, insulin resistance.

**Figure 9:**
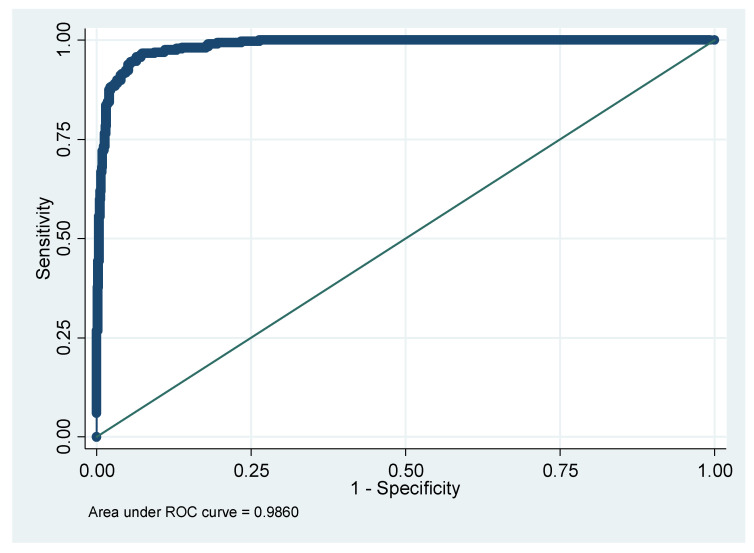
AUC for whether to have IR with VOCs, demographic biochemistry, and lifestyle model. Note: AUC, the area under the ROC curve; IR, insulin resistance; VOCs, volatile organic compounds.

Finally, the results of the logistic regression analysis are shown in Table 7. The area under the ROC curve (AUC), sensitivity, specificity, PPV, and NPV are presented. Notably, when using only VOCs without other features, AUC reached up to 0.8863 (Figure 7). At the same time, if only the demographic, biochemistry, and lifestyle are considered, the AUC is only 0.8484 (Figure 8). After adding VOCs, AUC increased to 0.9860 (Figure 9).

## 4. Discussion

IR is widely recognized for its importance in the development of T2D [29]. The present study focuses on the impact of VOCs. However, evaluating the influence of VOCs on IR requires the consideration of confounding factors; thus, our analysis includes demographic, biochemistry, and lifestyle factors. Table 6 shows the various impacts of WC, TG, HDL-C, liver enzymes, UA, SBP, marital status, and age on HOMA-IR, with results consistent with previous findings [29,30,31]. Both MLR and Mach-L can simultaneously evaluate and adjust all confounding factors. Our results showed that the most important impacting factor was waist circumference, with a 100% importance value. Dodecane was the seventh most important factor, and the other five VOCs were all among the top 16 factors in terms of importance, indicating potential for use in detecting IR. Here, we only focus on the novice roles of VOCs and IR. It could be argued that these VOCs have less impact on the HOMA-IR. However, these VOCs have important roles for IR and should be studied further in the future.

Dodecane and aniline show opposite directions from simple correlation and net SHAP values, and we are currently unable to explain the discrepancy, though it may have something to do with Mach-L operating by capturing non-linear relationships in a dataset. Secondly, there are discrepancies between the results of simple correlation and SHAP. This is because, contrary to simple correlation, SHAP examines the interactions between all the parameters. This is the reason for the different results from simple correlation and SHAP.

One might argue that the features selected by the SHAP KernelExplainer differ from those identified by SHAP. This discrepancy can be explained by the use of different algorithms. The SHAP KernelExplainer is model-agnostic and uses a sampling-based approximation of SHAP values. It assumes independence among features and does not consider model internals. We used this method to support the interpretation and accuracy of SHAP results.

To further evaluate the effects of VOCs on IR, we performed logistic regression. The results showed that using only VOCs, AUC reached 0.8863. After adding other features (demographic, biochemical, and lifestyle), the AUC increased to 0.9860. These findings suggest that VOCs play an important role in IR. The most impactful VOC was found to be dodecane, a solvent used in delivering phospholipids to cells, which had not been previously reported as being relevant to IR. The ethanol/dodecane system can induce cytotoxic effects and influence cellular processes, but these effects are not specifically attributed to inflammation [32,33]. At the same time, the ceramide-1-phosphate delivered by dodecane stimulates prostaglandins, which are a well-known inflammatory marker [33,34]. While there is no direct evidence for a connection, this finding suggests dodecane is related to inflammation, which is the key for developing IR [35,36]. The present study is thus the first to propose this possible relationship between dodecane and IR, and further study is needed.

The second most important VOC was dimethylfuran, a compound used as a potential biofuel due to its high energy density and stability [37]. Some furan derivatives are known to generate reactive oxygen species (ROS), which can contribute to oxidative stress. Oxidative stress is a key factor in the development of IR [38]. Chronic inflammation is another contributor to IR. If dimethylfuran or its metabolites promote inflammatory pathways, it could indirectly affect insulin sensitivity [39,40]. This evidence could support the findings of the present study.

The third most impactful VOC is butoxyethanol. The relationship between butoxyethanol and IR has not been extensively documented, but exposure to butoxyethanol may influence metabolic processes that could contribute to IR. Butoxyethanol, also known as ethylene glycol monobutyl ether, is a solvent commonly used in various industrial applications and household products. It has been studied for its potential toxicological effects, particularly in terms of its deleterious impact on the liver and metabolic health. The liver plays a crucial role in glucose metabolism and insulin sensitivity. Damage to liver cells can impair insulin signaling pathways, potentially leading to IR over time [1,41]. Exposure to butoxyethanol could also be associated with increased oxidative stress, which is also a known contributor to IR [42,43]. This novel finding of the potential impact of VOCs could provide new insight into the role of butoxyethanol and IR.

The next most-impactful VOCs is propanamine, a compound that has been shown to beneficially enhance glucose consumption by insulin-resistant cells, thereby reducing the incidence and severity of IR. These compounds can reduce blood glucose levels in T2D models by improving carbohydrate tolerance and reducing triglyceride levels [44]. This relationship could be explained by the following mechanisms:

1. Glucose utilization: Propanamine compounds increase the glucose uptake in insulin-resistant cells [45,46].
2. Lipid profile improvement: These compounds also positively affect lipid profiles by decreasing TG and LDL-C while increasing HDL-C levels [45].

The fifth most important VOCs was aniline, which has been shown to induce oxidative stress in various cell types, including hepatocytes. This oxidative stress is characterized by increased levels of reactive oxygen species (ROS) and lipid peroxidation, which can lead to cellular damage and apoptosis [47]. Oxidative stress plays a crucial role in the development of IR. Elevated ROS levels can impair insulin signaling pathways, leading to reduced glucose uptake by cells and contributing to metabolic disorders such as T2D [48]. Aniline’s ability to increase oxidative stress may therefore exacerbate IR by damaging pancreatic beta cells, which are essential for insulin production and secretion. Our results are consistent with these previous findings.

Finally, isopropyltoluene is an aromatic hydrocarbon primarily used as a solvent and in chemical synthesis, but little is known in terms of its effects on human health, particularly concerning metabolic disorders like IR. Aromatic hydrocarbons, including isopropyltoluene, may disrupt normal metabolic processes. Exposure to other similar compounds has been linked to alterations in glucose metabolism and insulin signaling [49,50]. In a review article, Arciola et al. highlighted its biological activity, which may include interactions with metabolic pathways [51]. While there is no direct evidence that toluene could disrupt glucose metabolism and insulin signaling, Dick et al. showed that chronic inhalation of toluene might have untoward effects on energy balance and glucose control [52]. Research examining the interaction between glucose and toluene metabolism in bacteria found that toluene influenced glucose metabolism, particularly affecting the glucokinase pathway, indicating that toluene can disrupt glucose metabolic processes at the cellular level [53]. These findings support our results for the potential relationship between isopropyltoluene and IR.

Our study is subject to certain limitations. First, the results of simple correlation (Table 4) and the net values of SHAP (Figure 5) show different directions (positive or negative) for dodecane and aniline. This discrepancy is difficult to explain, but may result from the complexity of both the Mach-L models and VOCs, and further studies with more detailed designs are needed. Secondly, this is a cross-sectional study, which is less convincing than a longitudinal one. Thirdly, we did not exclude participants using medications that might affect examined variables such as blood pressure, blood glucose, and lipids, potentially leading to secondary effects in our VOCs results. Fourthly, due to the relatively limited sample size, the entire dataset was used for model construction and AUC analysis as part of an initial exploratory evaluation. Future studies may consider applying cross-validation or independent test datasets to enhance model robustness and generalizability. Finally, the sample for the present study was limited to a single ethnic group, and extrapolation to other ethnic groups should be performed with caution.

## 5. Conclusions

Using three different Mach-L methods, six VOCs were found to be related to IR in Taiwanese women. In decreasing order of importance, they are dimethylfuran, propanamine, aniline, butoxyethanol, and isopropyltoluene. Furthermore, SHAP was used to indicate the positive and negative impacts of these VOCs, where dimethylfuran, isopropyltoluene, and dodecane had positive effects, and the other three had negative effects. These findings suggest VOCs could potentially be used to effectively detect HOMA-IR.

## Figures and Tables

**Table 1:** Demographic data of participants.

Characteristic	Mean ± SD
N number	1432
Age (yr)	44.98 ± 12.27
Waist circumference (cm)	73.10 ± 8.36
Systolic blood pressure (mmHg)	108.97 ± 15.51
Diastolic blood pressure (mmHg)	70.13 ± 9.61
Serum glutamic oxaloacetic transaminase (IU/L)	20.98 ± 8.75
Serum glutamic pyruvic transaminase (IU/L)	20.44 ± 15.48
Estimated Glomerular filtration rate (mL/min/1.73 m^2^)	86.61 ± 13.89
Uric acid (mg/dL)	4.83 ± 1.02
Triglyceride (mg/dL)	85.25 ± 56.32
High-density lipoprotein cholesterol (mg/dL)	62.37 ± 15.56
Low-density lipoprotein cholesterol (mg/dL)	114.17 ± 31.01
Homeostasis Model Assessment for Insulin Resistance	1.61 ± 1.31
Alcohol consumption	2.33 ± 11.09
Tobacco consumption	1.11 ± 6.30
Betel nut consumption	0.00 ± 0.00
Exercise habits	5.41 ± 7.39
Sleep habits	2.97 ± 0.86
Marital status, *n* (%)	
Unmarried	519 (38.91)
Married	815 (61.09)
Education, *n* (%)	
(1) No formal schooling	3 (0.23)
(2) Elementary school	24 (1.82)
(3) Junior high school	35 (2.66)
(4) High school (vocational)	207 (15.72)
(5) Junior college	230 (17.46)
(6) University	601 (45.63)
(7) Graduate school or above	217(16.48)
Annual Income level (IL) (TWD)	
(1) Below USD200,000	191 (15.09)
(2) USD200,001–USD400,000	173 (13.67)
(3) USD400,001–USD800,000	478 (37.76)
(4) USD800,001–USD1,200,000	250 (19.75)
(5) USD1200,001–USD1,600,000	90 (7.11)
(6) USD1,600,001–USD2,000,000	40 (3.16)
(7) More than USD2,000,000	44 (3.48)

**Table 2:** Performance metrics equations.

Metrics	Description	Calculation
MAPE	Mean Absolute Percentage Error	$MAPE=\frac{1}{n}\sum_{i=1}^{n}\left|\frac{y_{i}-{\hat{y}}_{i}}{y_{i}}\right|\times 100$
SMAPE	Symmetric Mean Absolute Percentage Error	$SMAPE=\frac{1}{n}\sum_{i=1}^{n}\frac{\left|y_{i}-{\hat{y}}_{i}\right|}{\left(\left|y_{i}\right|+\left|{\hat{y}}_{i}\right|\right)/2}\times 100$
RAE	Relative Absolute Error	$RAE=\sqrt{\frac{\sum_{i=1}^{n}{\left(y_{i}-{\hat{y}}_{i}\right)}^{2}}{\sum_{i=1}^{n}{\left(y_{i}\right)}^{2}}}$
RRSE	Root Relative Squared Error	$RRSE=\sqrt{\frac{\sum_{i=1}^{n}{\left(y_{i}-{\hat{y}}_{i}\right)}^{2}}{\sum_{i=1}^{n}{\left(y_{i}-{\hat{y}}_{i}\right)}^{2}}}$
RMSE	Root Mean Squared Error	$RMSE=\sqrt{\frac{1}{n}\sum_{i=1}^{n}{\left(y_{i}-{\hat{y}}_{i}\right)}^{2}}$

Note: ${\hat{y}}_{i}$ and *y_i_* represent predicted and actual values, respectively; *n* stands the number of instances.

**Table 3:** Comparison of independent variables in participants with and without IR.

Characteristic	Without IR Mean ± SD	With IR Mean ± SD	*p*-Value
N number	1036	396	
Age (yr)	44.39 ± 11.74	46.49 ± 13.44	0.003
Waist circumference (cm)	70.58 ± 6.64	79.66 ± 8.82	0.000
Systolic blood pressure (mmHg)	106.66 ± 14.78	114.99 ± 15.78	0.000
Diastolic blood pressure (mmHg)	68.97 ± 9.36	73.13 ± 9.58	0.000
Serum glutamic oxaloacetic transaminase (IU/L)	20.41 ± 7.38	22.44 ± 11.47	0.000
Serum glutamic pyruvic transaminase (IU/L)	18.08 ± 11.95	26.61 ± 21.00	0.000
Estimated Glomerular filtration rate (mL/min/1.73 m^2^)	86.54 ± 13.46	86.77 ± 14.94	0.776
Uric acid (mg/dL)	4.65 ± 0.95	5.27 ± 1.04	0.000
Triglyceride (mg/dL)	72.72 ± 37.64	118.01 ± 79.31	0.000
High-density lipoprotein cholesterol (mg/dL)	65.48 ± 15.22	54.20 ± 13.31	0.000
Low-density lipoprotein cholesterol (mg/dL)	111.00 ± 29.35	122.44 ± 33.63	0.000
Homeostasis Model Assessment for Insulin Resistance	1.07 ± 0.33	2.99 ± 1.80	0.000
Alcohol consumption	2.55 ± 11.96	1.74 ± 8.39	0.217
Tobacco consumption	1.15 ± 6.45	0.99 ± 5.89	0.676
Betel nut consumption	0.00 ± 0.00	0.00 ± 0.00	-
Exercise habits	5.99 ± 7.89	3.87 ± 5.62	0.000
Sleep habits	2.98 ± 0.84	2.92 ± 0.88	0.222
Marital status, *n* (%)			
Unmarried	371 (38.57)	148 (39.78)	0.682
Married	591 (61.43)	224 (60.22)	
Education, *n* (%)			
(1) No formal schooling	2 (0.21)	1 (0.27)	0.003
(2) Elementary school	13 (1.36)	11 (3.02)	
(3) Junior high school	18 (1.89)	17 (4.67)	
(4) High school (vocational)	136 (14.27)	71 (19.51)	
(5) Junior college	169 (17.73)	61 (16.76)	
(6) University	452 (47.43)	149 (40.93)	
(7) Graduate school or above	163 (17.10)	54 (14.84)	
Annual Income level (IL) (TWD)			
(1) Below USD200,000	129 (14.07)	62 (17.77)	0.423
(2) USD200,001–USD400,000	120 (13.09)	53 (15.19)	
(3) USD400,001–USD800,000	352 (38.39)	126 (36.10)	
(4) USD800,001–USD1,200,000	190 (20.72)	60 (17.19)	
(5) USD1,200,001–USD1,600,000	68 (7.42)	22 (6.30)	
(6) USD1,600,001–USD2,000,000	28 (3.05)	12 (3.44)	
(7) More than USD2,000,000	30 (3.27)	14 (4.01)	

**Table 4:** The *r* values of Pearson’s correlation between HOMA-IR and VOCs, demographic, biochemistry, and lifestyle parameters.

	Dimethylfuran		Isopropyltoluene		Dodecane	
HOMA-IR	0.004		0.000		0.050	
	butoxyethanol		aniline		propanamine	
HOMA-IR	−0.023		−0.010		−0.021	
	age	WC	GOT	GPT	eGFR	UA
HOMA-IR	0.090 **	0.515 ***	0.224 ***	0.326 ***	−0.015	0.270 ***
	TG	HDL-C	LDL-C	SBP	DBP	
HOMA-IR	0.454 ***	−0.349 ***	0.173 ***	0.248 ***	0.186 ***	

Note: HOMA-IR, Homeostasis Model Assessment for Insulin Resistance; WC, waist circumference; GOT, serum glutamic oxaloacetic transaminase; GPT, serum glutamic pyruvic transaminase; eGFR, estimated Glomerular filtration rate; UA, uric acid; TG, triglycerides; HDL-C, high-density lipoprotein cholesterol; LDL-C, low-density lipoprotein cholesterol; SBP, systolic blood pressure; DBP, diastolic blood pressure. ** < 0.01, *** < 0.001.

**Table 5:** The estimation errors of MLR and three different Mach-L methods.

	MAPE	SMAPE	RAE	RRSE	RMSE
MLR	0.8003	0.5802	1.2768	1.29	1.6983
SGB	0.402	0.3311	0.7237	0.7489	0.986
XGBoost	0.4225	0.3334	0.7407	0.7508	0.9884
EN	0.4368	0.3458	0.7524	0.7757	1.0212

Note: MLR, multiple linear regression; SGB, stochastic gradient boosting; XGBoost, eXtreme gradient boosting; EN, elastic net; MAPE, mean absolute percentage error; SMAPE, symmetric mean absolute; RAE, relative absolute error; RRSE, root relative squared error; RMSE, root mean squared error.

**Table 6:** The percentage of importance of MLR, three different Mach-L methods, and the mean of the three Mach-L methods.

		MLR	SGB	XGBoost	EN	Mean
1	WC	100	100	100	100	100
2	TG	73.79	49.07	63.83	10.15	47.45
3	HDL-C	35.67	56.84	34.54	15.33	34.535
4	GPT	30.42	40.86	25.37	24.57	28.6525
5	GOT	3.62	8.95	4.52	0	7.9325
6	LDL-C	4.67	3.24	5.14	0	6.39
7	Dodecane	30.79	0.86	5.09	0	4.8775
8	UA	6.19	4.23	1.26	9.16	4.62
9	SBP	17.1	2.62	2.29	3.22	2.9225
10	Dimethylfuran	33.34	8.65	0	0	2.8425
11	Marital status	11.69	0	0	7.62	2.0775
12	Propanamine	0.66	3.64	3.59	0	2.0575
13	Aniline	10.6	3.37	0	0	1.745
14	Age	30.51	0	1.06	0	1.55
15	Butoxyethanol	18.09	4.8	0	0	1.445
16	Isopropyltoluene	8.7	1.51	0	0	1.4325

Note: MLR, multiple linear regression; SGB, stochastic gradient boosting; XGBoost, eXtreme gradient boosting; EN, elastic net; WC, waist circumference; TG, triglycerides; HDL-C, high-density lipoprotein cholesterol; GPT, serum glutamic pyruvic transaminase; GOT, serum glutamic oxaloacetic transaminase; LDL-C, low-density lipoprotein cholesterol; UA, uric acid; SBP, systolic blood pressure.

**Table 7:** The results of logistic regression of whether to have IR in subjects only with VOCs or with all other independent variables (demographic biochemistry, and lifestyle information).

	AUC	Sensitivity	Specificity	PPV	NPV
Model 1	0.8863	60.87%	92.56%	75.80%	86.07%
Model 2	0.8484	51.80%	92.93%	73.93%	83.28%
Model 3	0.9860	89.97%	96.47%	90.80%	96.13%

Note: Model 1: Only with VOCs; Model 2: Only with demographic biochemistry and lifestyle information; Model 3: both features from model 1 and 2. AUC: Area under the receiver operation curve; PPV, positive predictive value; NPV, negative predictive value.

## Data Availability

Data available on request due to privacy/ethical restrictions.

## Appendix A

**Table A1:** Three hundreds and forty four VOCs.

	**Name**	**Molecular Formula**
1	2,5-dimethylpyrazine (123-32-0)	C_6_H_8_N_2_ = 108.14
2	2,5-dimethylfuran (625-86-5)	C_6_H_8_O = 96.13
3	2-methylpyrazine (109-08-0)	C_5_H_6_N_2_ = 94.12
4	acetophenone (98-86-2)	C_8_H_8_O = 120.15
5	benzoic acid (65-85-0)	C_7_H_6_O_2_ = 122.12
6	beta-caryophyllene (87-44-5)	C_15_H_24_ = 204.36
7	4-isopropyl toluene (99-87-6)	C_10_H_14_ = 134.22
8	pyrrole (109-97-7)	C_4_H_5_N = 67.09
9	(E)-2-nonenal (18829-56-6)	C_9_H_16_O = 140.23
10	furfural (98-01-1)	C_5_H_4_O_2_ = 96.09
11	propyne (74-99-7)	C_3_H_4_ = 40.07
12	dodecane (112-40-3)	C_12_H_26_ = 170.34
13	6-methyl-5-hepten-2-one (110-93-0)	C_8_H_14_O = 126.20
14	pyridine (110-86-1)	C_5_H_5_N = 79.10
15	benzene (71-43-2)	C_6_H_6_ = 78.11
16	(E)-2-hexenal (6728-26-3)	C_6_H_10_O = 98.15
17	2-hexanone (591-78-6)	C_6_H_12_O = 100.16
18	1,3-butadiene (106-99-0)	C_4_H_6_ = 54.09
19	(E)-2-heptenal (18829-55-5)	C_7_H_12_O = 112.17
20	eucalyptol (470-82-6)	C_10_H_18_O = 154.25
21	1-butyne (107-00-6)	C_4_H_6_ = 54.09
22	2-octanone (111-13-7)	C_8_H_16_O = 128.22
23	octanal (124-13-0)	C_8_H_16_O = 128.22
24	tridecane (629-50-5)	C_13_H_28_ = 184.37
25	1,4-butyrolactone (96-48-0)	C_4_H_6_O_2_ = 86.09
26	styrene (100-42-5)	C_8_H_8_ = 104.15
27	alpha-terpinene (99-86-5)	C_10_H_16_ = 136.24
28	2-methylpentane (107-83-5)	C_6_H_14_ = 86.18
29	butyl 2-methylbutanoate (15706-73-7)	C_9_H_18_O_2_ = 158.24
30	3-pentanone (96-22-0)	C_5_H_10_O = 86.13
31	2-methyl-3-mercaptofuran (28588-74-1)	C_5_H_6_OS = 114.16
32	diethylethanolamine (100-37-8)	C_6_H_15_NO = 117.18
33	ethylcyclohexane (1678-91-7)	C_8_H_16_ = 112.22
34	1,2-propanediol (57-55-6)	C_3_H_8_O_2_ = 76.10
35	undecane (1120-21-4)	C_11_H_24_ = 156.31
36	oct-2-en-1-ol (18409-17-1)	C_8_H_16_O = 128.22
37	decane (124-18-5)	C_10_H_22_ = 142.29
38	1-methyl-2-pyrrolidinone (872-50-4)	C_5_H_9_NO = 99.13
39	3-methyl-2-butenal (107-86-8)	C_5_H_8_O = 84.12
40	norfuraneol (19322-27-1)	C_5_H_6_O_3_ = 114.10
41	4-methyloctanoic acid (54947-74-9)	C_9_H_18_O_2_ = 158.24
42	3-methyl-2-butanone (563-80-4)	C_5_H_10_O = 86.13
43	cyclohexanone (108-94-1)	C_6_H_10_O = 98.15
44	1-octen-3-ol (3391-86-4)	C_8_H_16_O = 128.22
45	propyl hexanoate (626-77-7)	C_9_H_18_O_2_ = 158.24
46	diallyl sulfide (592-88-1)	C_6_H_10_S = 114.21
47	indole (120-72-9)	C_8_H_7_N = 117.15
48	nonalactone gamma (104-61-0)	C_9_H_16_O_2_ = 156.23
49	2,3-dimethylheptane (3074-71-3)	C_9_H_20_ = 128.26
50	methylcyclopentane (96-37-7)	C_6_H_12_ = 84.16
51	1,5-pentanediol (111-29-5)	C_5_H_12_O_2_ = 104.15
52	piperidine (110-89-4)	H_8_N_6_ = 92.10
53	2-methylbutanal (96-17-3)	C_5_H_10_O = 86.13
54	2-methoxyethanol (109-86-4)	C_3_H_8_O_2_ = 76.10
55	3-buten-2-one (78-94-4)	C_4_H_6_O = 70.09
56	1-penten-3-one (1629-58-9)	C_5_H_8_O = 84.12
57	3-methylbutanal (590-86-3)	C_5_H_10_O = 86.13
58	pentanal (110-62-3)	C_5_H_10_O = 86.13
59	methyl butanoate (623-42-7)	C_5_H_10_O_2_ = 102.13
60	butanal (123-72-8)	C_4_H_8_O = 72.11
61	2-vinylpyridine (100-69-6)	C_7_H_7_N = 105.14
62	dipropenyl sulfide (627-51-0)	C_4_H_6_S = 86.16
63	2-decanone (693-54-9)	C_10_H_20_O = 156.26
64	2-pentanone new (107-87-9)	C_5_H_10_O = 86.13
65	2-methyl-3-pentanone (565-69-5)	C_6_H_12_O = 100.16
66	(E)-2-hexene (4050-45-7)	C_6_H_12_ = 84.16
67	heptanal (111-71-7)	C_7_H_14_O = 114.19
68	thiazole (288-47-1)	C_3_H_3_NS = 85.12
69	2-methylpropanal (78-84-2)	C_4_H_8_O = 72.11
70	dimethoxymethane (109-87-5)	C_3_H_8_O_2_ = 76.10
71	butyl propanoate (590-01-2)	C_7_H_14_O_2_ = 130.19
72	ethyl isopropyl amine (19961-27-4)	C_5_H_13_N = 87.17
73	decanal (112-31-2)	C_10_H_20_O = 156.27
74	1,4-thioxane (15980-15-1)	C_4_H_8_OS = 104.17
75	methacrylic acid (79-41-4)	C_4_H_6_O_2_ = 86.09
76	2-hexene (592-43-8)	C_6_H_12_ = 84.16
77	Z-2-hexene (7688-21-3)	C_6_H_12_ = 84.16
78	3-methyl-2-butanol (598-75-4)	C_5_H_12_O = 88.15
79	2-butene (107-01-7)	C_47_H_51_NO_14_ = 853.92
80	(S)-2-methyl-1-butanol (1565-80-6)	C_5_H_12_O = 88.15
81	2-methylbutanoic acid (116-53-0)	C_5_H_10_O_2_ = 102.13
82	isobutyl butanoate (539-90-2)	C_8_H_16_O_2_ = 144.21
83	propylbenzene (103-65-1)	C_9_H_12_ = 120.20
84	2-octene (111-67-1)	C_8_H_16_ = 112.22
85	(E)-2-octene (13389-42-9)	C_8_H_16_ = 112.22
86	3,3-dimethyl-2-butanone (75-97-8)	C_6_H_12_O = 100.16
87	propyl propanoate (106-36-5)	C_6_H_12_O_2_ = 116.16
88	3-methyl-3-buten-1-ol (763-32-6)	C_5_H_10_O = 86.13
89	3-methylbutanoic acid (503-74-2)	C_5_H_10_O_2_ = 102.13
90	2-methyl-1-butanol (137-32-6)	C_5_H_12_O = 88.15
91	limonene (138-86-3; 7705-14-8)	C_10_H_16_ = 136.23
92	methyl diethanolamine (105-59-9)	C_5_H_13_NO_2_ = 119.16
93	benzaldehyde (100-52-7)	C_7_H_6_O = 106.12
94	nonanal (124-19-6)	C_9_H_18_O = 142.24
95	methyl methacrylate (80-62-6)	CH_2_ = 100.12
96	methyl isobutyrate (547-63-7)	C_5_H_10_O_2_ = 102.13
97	cyclohexanol (108-93-0)	C_6_H_11_OH = 100.16
98	(Z)-2-octene (7642-04-8)	C_8_H_16_ = 112.22
99	nitromethane (75-52-5)	CH_3_NO_2_ = 61.04
100	1-methoxybutane (628-28-4)	C_5_H_12_O = 88.15
101	tetrahydropyrrole (123-75-1)	C_4_H_9_N = 71.12
102	(S)-2-pentanol (26184-62-3)	C_5_H_12_O = 88.15
103	(R)-2-pentanol (31087-44-2)	C_5_H_12_O = 88.15
104	2-pentanol (6032-29-7)	C_5_H_12_O = 88.15
105	allyl cyanide (109-75-1)	C_4_H_5_N = 67.09
106	terpinolene (586-62-9)	C_10_H_16_ = 136.24
107	cyclopropanecarboxylic acid (1759-53-1)	C_4_H_6_O_2_ = 86.09
108	1,3-propanediol (504-63-2)	C_3_H_8_O_2_ = 76.10
109	beta-propiolactone (57-57-8)	C_3_H_4_O_2_ = 72.06
110	3-methylthiophene (616-44-4)	C_5_H_6_S = 98.16
111	dimethyl isopropyl amine (996-35-0)	C_5_H_13_N = 87.17
112	3-pentanol (584-02-1)	C_5_H_12_O = 88.15
113	2-methyl-2-butanol (75-85-4)	C_5_H_12_O = 88.15
114	3-methyl-1-butanol (123-51-3)	C_5_H_12_O = 88.15
115	1-pentanol (71-41-0)	C_5_H_12_O = 88.15
116	methanamide (75-12-7)	CH_3_NO = 45.04
117	ethanolamine (141-43-5)	C_2_H_7_NO = 61.08
118	1,2,4-trimethylbenzene (95-63-6)	C_9_H_12_ = 120.20
119	3-methylpentane (96-14-0)	C_6_H_14_ = 86.18
120	ethyl propanoate (105-37-3)	C_5_H_10_O_2_ = 102.13
121	2-propyl acetate (108-21-4)	C_5_H_10_O_2_ = 102.13
122	acrylamide (79-06-1)	C_3_H_5_NO = 71.08
123	allyl ethyl ether (557-31-3)	C_5_H_10_O = 86.13
124	methyl acrylate (96-33-3)	C_4_H_6_O_2_ = 86.09
125	pentanoic acid (109-52-4)	C_5_H_10_O_2_ = 102.13
126	(+/−)-2-pentanamine (63493-28-7)	C_5_H_13_N = 87.17
127	(S)-2-octanol (6169-06-8)	C_8_H_18_O = 130.23
128	(R)-2-octanol (5978-70-1)	C_8_H_18_O = 130.23
129	2-methoxy-2-methyl butane (994-05-8)	C_6_H_14_O = 102.17
130	methyl hexanoate (106-70-7)	C_7_H_14_O_2_ = 130.19
131	3-methyl-2-buten-1-ol (556-82-1; 60766-00-9)	C_5_H_10_O = 86.13
132	1-octanol (111-87-5)	C_8_H_18_O = 130.23
133	2,2-dimethyl-1-propanol (75-84-3)	C_5_H_12_O = 88.15
134	cyclohexanamine (108-91-8)	C_6_H_13_N = 99.18
135	2-hexen-1-ol (2305-21-7)	C_6_H_12_O = 100.160
136	3-methylthio-2-butanone (53475-15-3)	C_5_H_10_OS = 118.19
137	isobutene (115-11-7)	C_4_H_8_ = 56.11
138	trans-2-penten-1-ol (1576-96-1)	C_2_H_5_CH = 86.13
139	dimethylacetamide (127-19-5)	C_4_H_9_NO = 87.12
140	3-methyl-2-butanamine (598-74-3)	C_5_H_13_N = 87.16
141	2-thioethanol (60-24-2)	C_2_H_6_OS = 78.13
142	isopropyl methyl sulfide (1551-21-9)	C_4_H_10_S = 90.18
143	2-octanol (123-96-6; 4128-31-8)	C_8_H_18_O = 130.23
144	cis-2-penten-1-ol (1576-95-0)	C_5_H_10_O = 86.13
145	methyl diethyl amine (616-39-7)	C_5_H_13_N = 87.17
146	2-penten-1-ol (20273-24-9)	C_5_H_10_O = 86.13
147	propyl butanoate (105-66-8)	C_7_H_14_O_2_ = 130.19
148	2-pentanamine (625-30-9)	C_5_H_13_N = 87.16
149	methyl pentanoate (624-24-8)	C_6_H_12_O_2_ = 116.16
150	tertiary butyl acetate (540-88-5)	C_6_H_12_O_2_ = 116.16
151	1,5-pentanedial (111-30-8)	C_5_H_8_O_2_ = 100.12
152	methyl butyl amine (110-68-9)	C_5_H_13_N = 87.17
153	1-octene (111-66-0)	C_8_H_16_ = 112.22
154	(Z)-2-hexen-1-ol (928-94-9)	C_6_H_12_O = 100.16
155	1,5-diaminopentane (462-94-2)	C_5_H_14_N_2_ = 102.18
156	2-methyl-2-propenal (78-85-3)	C_4_H_6_O = 70.09
157	(Z)-2-pentene (627-20-3)	C_5_H_10_ = 70.14
158	3-methyltetrahydrofuran-3-one (3188-00-9)	C_5_H_8_O_2_ = 100.12
159	isopropyl propanoate (637-78-5)	C_6_H_12_O_2_ = 116.16
160	2-methyl-2-butene (513-35-9)	C_5_H_10_ = 70.14
161	2,3-butanedione (431-03-8)	C_4_H_6_O_2_ = 86.09
162	2-methyl-1-butanamine (96-15-1)	C_5_H_13_N = 87.17
163	butanal oxime (110-69-0)	C_4_H_9_NO = 87.12
164	butanone (78-93-3)	C_4_H_8_O = 72.11
165	1-pentanamine (110-58-7)	C_5_H_13_N = 87.17
166	2-pentene (109-68-2)	C_5_H_10_ = 70.14
167	s-butylamine (13952-84-6)	C_4_H_11_N = 73.14
168	3-mercapto-3-methylbutyl formate (50746-10-6)	C_6_H_12_O_2_S = 148.22
169	diisopropyl ether (108-20-3)	C_6_H_14_O = 102.18
170	butyl methanoate (592-84-7)	C_5_H_10_O_2_ = 102.13
171	hexanal (66-25-1)	C_6_H_12_O = 100.16
172	1-penten-3-ol (616-25-1)	C_5_H_10_O = 86.13
173	(E)-2-pentene (646-04-8)	C_5_H_10_ = 70.14
174	ethyl butanoate (105-54-4)	C_6_H_12_O_2_ = 116.16
175	L-lactic acid (79-33-4)	C_3_H_6_O_3_ = 90.08
176	1-octen-3-one (4312-99-6)	C_8_H_14_O = 126.20
177	1,4-butanediol (110-63-4)	C_4_H_10_O_2_ = 90.12
178	1-heptene (592-76-7)	C_7_H_14_ = 98.19
179	isoamyl amine (107-85-7)	C_5_H_13_N = 87.17
180	1-butanamine (109-73-9)	C_4_H_11_N = 73.14
181	furfuryl alcohol (98-00-0)	C_5_H_6_O_2_ = 98.10
182	2-methyl-3-buten-2-ol (115-18-4)	C_5_H_10_O = 86.13
183	acrylic acid (79-10-7)	C_3_H_4_O_2_ = 72.06
184	(E)-2-hexen-1-ol (928-95-0)	C_6_H_12_O = 100.16
185	methyl n-propyl sulfide (3877-15-4)	C_4_H_10_S = 90.18
186	ethyl methanoate (109-94-4)	C_3_H_6_O_2_ = 74.08
187	gamma-terpinene (99-85-4)	C_10_H_16_ = 136.24
188	(S)-limonene (5989-54-8)	C_10_H_16_ = 136.24
189	dimethyl sulfate (77-78-1)	C_2_H_6_O_4_S = 126.13
190	dimethyl sulfide (75-18-3)	C_2_H_6_S = 62.13
191	2-phenylethanol (60-12-8)	C_8_H_10_O = 122.17
192	methyl 2-methylbutanoate (868-57-5)	C_6_H_12_O_2_ = 116.16
193	2,2-dimethyl propanoic acid (75-98-9)	C_5_H_10_O_2_ = 102.13
194	methyl acetate (79-20-9)	C_3_H_6_O_2_ = 74.08
195	isopropyl benzene (98-82-8)	C_9_H_12_ = 120.20
196	2-methyl-2-nitropropane (594-70-7)	C_4_H_9_NO_2_ = 103.12
197	octanoic acid (124-07-2)	C_8_H_16_O_2_ = 144.21
198	acrylonitrile (107-13-1)	C_3_H_3_N = 53.06
199	1-hexene (592-41-6)	C_6_H_12_ = 84.16
200	acetic anhydride (108-24-7)	C_4_H_6_O_3_ = 102.09
201	phenylacetic acid (103-82-2)	C_8_H_8_O_2_ = 136.15
202	propyl acetate (109-60-4)	C_5_H_10_O_2_ = 102.13
203	ethyl benzoate (93-89-0)	C_9_H_10_O_2_ = 150.18
204	2-propanethiol (75-33-2)	C_3_H_8_S = 76.16
205	2,3-pentanedione (600-14-6)	C_5_H_8_O_2_ = 100.12
206	1-butanethiol (109-79-5)	C_4_H_10_S = 90.18
207	propanoic acid (79-09-4)	C_3_H_6_O_2_ = 74.08
208	(R)-1-phenylethanol (1517-69-7)	C_8_H_10_O = 122.17
209	2,3-dimethylpentane (565-59-3)	C_7_H_16_ = 100.21
210	1-phenylethanol (98-85-1)	C_8_H_10_O = 122.17
211	alpha-pinene (80-56-8; 2437-95-8)	C_10_H_16_ = 136.23
212	beta-pinene (127-91-3)	C_10_H_16_ = 136.23
213	(S)-1-phenylethanol (1445-91-6)	C_8_H_10_O = 122.17
214	diethyl ether (60-29-7)	C_4_H_10_O = 74.12
215	3-butyn-2-ol new (2028-63-9)	C_4_H_6_O = 70.09
216	1R-(+)-alpha-pinene (7785-70-8)	C_10_H_16_ = 136.24
217	2-nitropropane (79-46-9)	C_3_H_7_NO_2_ = 89.09
218	ethyl mercaptan (75-08-1)	C_2_H_6_S = 62.13
219	isopentane (78-78-4)	C_5_H_12_ = 72.15
220	dipropenyl disulfide (629-19-6)	C_6_H_14_S_2_ =150.3
221	1,4-dioxane (123-91-1)	C_4_H_8_O_2_ = 88.11
222	butanoic acid (107-92-6)	C_4_H_8_O_2_ = 88.11
223	1-nitropropane (108-03-2)	C_3_H_7_NO_2_ = 89.09
224	3-pentanamine (616-24-0)	C_5_H_13_N = 87.17
225	1,4-diaminobutane (110-60-1)	C_4_H_12_N_2_ = 88.15
226	isobutyl alcohol (78-83-1)	C_4_H_10_O = 74.12
227	2-butoxyethanol (111-76-2)	C_6_H_14_O_2_ = 118.18
228	1-propanethiol (107-03-9)	C_3_H_8_S = 76.16
229	1-nonene (124-11-8)	C_9_H_18_ = 126.24
230	hexanoic acid (142-62-1)	C_6_H_12_O_2_ = 116.16
231	methionol (505-10-2)	C_4_H_10_OS = 106.18
232	methylhydrazine (60-34-4)	CH_6_N_2_ = 46.072
233	1-butanol (71-36-3)	C_4_H_10_O = 74.12
234	isopropylamine (75-31-0)	C_3_H_9_N = 59.11
235	3-mercapto-1-propanol (19721-22-3)	C_3_H_8_OS = 92.16
236	(S)-(+)-3-methylhexane (6131-24-4)	C_7_H_16_ = 100.20
237	acrolein (107-02-8)	C_3_H_4_O = 56.06
238	1,3-butanediol (107-88-0)	C_4_H_10_O_2_ = 90.12
239	aniline (62-53-3)	C_6_H_7_N = 93.13
240	1-butene (106-98-9)	C_7_H_5_ClO_3_ = 172.56
241	p-xylene (106-42-3)	C_8_H_10_ = 106.17
242	ethylbenzene (100-41-4)	C_8_H_10_ = 106.17
243	farnesol (4602-84-0)	C_15_H_26_O = 222.37
244	xylenes + ethylbenzene (1330-20-7)	C_8_H_10_ = 106.16
245	1,2-diaminocyclohexane (694-83-7)	C_6_H_14_N_2_ = 114.19
246	2-methyl-2-butanamine (594-39-8)	C_5_H_13_N = 87.17
247	trans-farnesol (4602-84-0)	C_15_H_26_O = 222.37
248	(RS)-1,3-butanediol (18826-95-4)	C_4_H_10_O_2_ = 90.12
249	vinyl acetate (108-05-4)	C_4_H_6_O_2_ = 86.09
250	beta-ocimene (13877-91-3)	C_10_H_16_ = 136.23
251	(E)-beta-ocimene (3779-61-1)	C_10_H_16_ = 136.23
252	beta-farnesol (58181-76-3)	C_15_H_24_O = 20.35
253	2-butanol (78-92-2)	C_4_H_10_O = 74.12
254	1-pentene (109-67-1)	C_5_H_10_ = 70.14
255	2,2-dimethylhexane (590-73-8)	C_8_H_18_ = 114.23
256	butyl hexanoate (626-82-4)	C_10_H_20_O_2_ = 172.27
257	3-methylhexane (589-34-4)	C_7_H_16_ = 100.21
258	isooctane (540-84-1)	C_8_H_18_ = 114.23
259	(Z)-beta-ocimene (3338-55-4)	C_10_H_16_ = 136.23
260	cis-alpha-ocimene (6874-44-8)	C_10_H_16_ = 136.23
261	propanal (123-38-6)	C_3_H_6_O = 58.08
262	nonane (111-84-2)	C_9_H_20_ = 128.26
263	isobutyl acetate (110-19-0)	C_6_H_12_O_2_ = 116.16
264	tertiary butyl propanoate (20487-40-5)	C_7_H_14_O_2_ = 130.19
265	urethane (51-79-6)	C_3_H_7_NO_2_ = 89.09
266	cyanoacetylene (1070-71-9)	C_3_HN = 51.05
267	2-methyl-2-propanethiol (75-66-1)	C_4_H_10_S = 90.18
268	octane (111-65-9)	C_8_H_18_ = 114.23
269	isobutanoic acid (79-31-2)	C_4_H_8_O_2_ = 88.11
270	3-vinylpyridine (1121-55-7)	C_7_H_7_N = 105.14
271	dipropyl sulfide (111-47-7)	C_6_H_14_S = 118.24
272	ethene (74-85-1)	CH_2_ = 28.05
273	1-phenyl-2-propanol (14898-87-4; 698-87-3)	C_9_H_12_O = 136.19
274	2-(dimethylamino)ethanol (108-01-0)	C_4_H_11_NO = 89.14
275	2-methyl-2-propanol (75-65-0)	C_4_H_10_O = 74.12
276	2-methylthioacetic acid (2444-37-3)	C_3_H_6_O_2_S = 106.15
277	butyl acetate (123-86-4)	C_6_H_12_O_2_ = 116.16
278	carbon disulfide (75-15-0)	CS_2_ = 76.13
279	[R,R]-2,3-butanediol (24347-58-8)	C_4_H_10_O_2_ = 90.12
280	(R,S)-butan-2,3-diol (5341-95-7)	C_4_H_10_O_2_ = 90.12
281	2,3-butanediol (513-85-9; 513-89-3)	C_4_H_10_O_2_ = 90.12
282	2,2-dimethylbutane (75-83-2)	C_6_H_14_ = 86.18
283	cyclohexane (110-82-7)	C_6_H_12_ = 84.16
284	propene (115-07-1)	C_3_H_6_ = 42.08
285	[S,S]-2,3-butanediol (19132-06-0)	C_4_H_10_O_2_ = 90.12
286	diethyl amine (109-89-7)	C_4_H_11_N = 73.14
287	1-methoxy-2-propanol (107-98-2)	C_4_H_10_O_2_ = 90.12
288	ethylene oxide (75-21-8)	C_2_H_4_O = 44.05
289	thiolacetic acid (507-09-5)	C_2_H_4_OS = 76.11
290	methyl isocyanate (624-83-9)	C_2_H_3_NO = 57.05
291	cyclopropane (75-19-4)	C_3_H_6_ = 42.08
292	methyl propanoate (554-12-1)	C_4_H_8_O_2_ = 88.11
293	2-butyl acetate (105-46-4)	C_6_H_12_O_2_ = 116.16
294	1,2-butanediol (584-03-2)	C_4_H_10_O_2_ = 90.12
295	cyclopentane (287-92-3)	C_5_H_10_ = 70.14
296	1-propanamine (107-10-8)	C_3_H_9_N = 59.11
297	2-ethoxyethanol (110-80-5)	C_4_H_10_O_2_ = 90.12
298	2-methyl-2-pentanamine (53310-02-4)	C_6_H_15_N = 101.19
299	ethyl propyl amine (20193-20-8)	C_5_H_13_N = 87.17
300	ethanamide (60-35-5)	C_2_H_5_NO = 59.07
301	acetic acid (64-19-7)	C_2_H_4_O_2_ = 60.05
302	methyl ethyl amine (624-78-2)	C_3_H_9_N = 59.11
303	1,2-dimethoxy ethane (110-71-4)	C_4_H_10_O_2_ = 90.12
304	thiolactic acid (79-42-5)	C_3_H_6_O_2_S = 106.14
305	acetonitrile (75-05-8)	C_2_H_3_N = 41.05
306	ethyldiethanolamine (139-87-7)	C_6_H_15_NO_2_ = 133.19
307	dimethyl ether (115-10-6)	C_2_H_6_O = 46.07
308	o-xylene (95-47-6)	C_8_H_10_ = 106.17
309	dimethylamine (124-40-3)	C_2_H_7_N = 45.09
310	furan (110-00-9)	C_4_H_4_O = 68.08
311	methylamine (74-89-5)	CH_5_N = 31.06
312	1,3-butadiyne (460-12-8)	C_4_H_2_ = 50.06
313	methyl methanoate (107-31-3)	C_2_H_4_O_2_ = 60.05
314	2-propanol (67-63-0)	C_3_H_8_O = 60.10
315	isobutane (75-28-5)	C_4_H_10_ = 58.12
316	ammonia (7664-41-7)	NH_3_ = 17.03
317	heptane (142-82-5)	C_7_H_16_ = 100.21
318	allyl methyl sulfide (10152-76-8)	C_4_H_8_S = 88.17
319	trimethylamine (75-50-3)	C_3_H_9_N = 59.11
320	acetaldehyde (75-07-0)	C_2_H_4_O = 44.05
321	pentane (109-66-0)	C_5_H_12_ = 72.15
322	isopropyl butanoate (638-11-9)	C_7_H_14_O_2_ = 130.19
323	formaldehyde (50-00-0)	CH_2_O = 30.03
324	propane (74-98-6)	C_3_H_8_ = 44.1
325	isoprene (78-79-5)	C_5_H_8_ = 68.12
326	toluene (108-88-3)	C_7_H_8_ = 92.14
327	acetoin (513-86-0)	C_4_H_8_O_2_ = 88.11
328	ethane (74-84-0)	C_6_H_5_NO_2_S = 155.17
329	ethyl acetate (141-78-6)	C_4_H_8_O_2_ = 88.11
330	hexane (110-54-3)	C_6_H_14_ = 86.18
331	1-propanol (71-23-8)	C_3_H_8_O = 60.10
332	butane (106-97-8)	C_4_H_10_ = 58.12
333	pyruvic acid (127-17-3)	C_3_H_4_O_3_ = 88.06
334	hydrogen peroxide (7722-84-1)	H_2_O_2_ = 34.01
335	2-mercapto-3-butanol (37887-04-0)	C_4_H_10_OS = 106.19
336	acetone (67-64-1)	C_3_H_6_O = 58.08
337	formic acid (64-18-6)	CH_2_O_2_ = 46.03
338	ethyl methyl sulfide (624-89-5)	C_3_H_8_S = 76.16
339	ethanol (64-17-5)	C_2_H_6_O = 46.07
340	methanol (67-56-1)	CH_4_O = 32.04
341	propylene oxide (75-56-9)	C_3_H_6_O = 58.08
342	ethanedial (107-22-2)	C_2_H_2_O_2_ = 58.04
343	nitric oxide (10102-43-9)	NO = 30.00
344	methane (74-82-8)	CH_4_ = 16.04
